# Topical Corticosteroid-Related Concerns and Phobic Behaviors in Saudi Arabia: A Cross-Sectional Investigation

**DOI:** 10.3390/healthcare14111461

**Published:** 2026-05-25

**Authors:** Mohammed K. Alghamdi, Rena H. Alharbi, Yunus M. Al-Zahrani, Khadija T. Habib, Samaa A. Sindi, Mohammad S. Alghamdi, Anwar Ali Alshehri, Manar AlAli, Abdullah S. Algarni, Mohammad A. Jareebi, Radwan A. Abutaleb, Mostafa Mohrag, Sameer Alqassimi, Ghazi I. Al Jowf, Mutaz M. Zogail

**Affiliations:** 1 Department of Dermatology, King Fahd Hospital, Jeddah 23325, Saudi Arabia; mkih.ghamdi@gmail.com; 2 Faculty of Medicine, Jazan University, Jazan 45142, Saudi Arabia; moataz00999@gmail.com; 3 Department of Dermatology, Riyadh First Health Cluster, King Saud Medical City, Riyadh 12746, Saudi Arabia; alzahraniyounes@gmail.com; 4 Faculty of Medicine, Umm Al-Qura University, Makkah 24382, Saudi Arabia; k.t.habib@hotmail.com (K.T.H.); samaa_sindi@outlook.sa (S.A.S.); 5 Department of Internal Medicine, Heraa General Hospital, Jeddah 22233, Saudi Arabia; mosugmd2008@gmail.com; 6 College of Medicine, University of Bisha, Bisha 67714, Saudi Arabia; ianwar.alshehri@gmail.com; 7 King Faisal General Hospital, Hofuf 36361, Saudi Arabia; manarsami44@gmail.com; 8 Department of Medicine, College of Medicine, University of Jeddah, Jeddah 22231, Saudi Arabia; dr-asg-86@hotmail.com; 9 Department of Family and Community Medicine, College of Medicine, Jazan University, Jazan 45142, Saudi Arabia; 10 Department of Dermatology, Jazan General Hospital, Jazan 45142, Saudi Arabia; radwana@moh.gov.sa; 11 Department of Internal Medicine, College of Medicine, Jazan University, Jazan 45142, Saudi Arabia; mmohrag@jazanu.edu.sa (M.M.); smalqassimi@jazanu.edu.sa (S.A.); 12 Department of Public Health, College of Applied Medical Sciences, University Medical Clinics Complex, King Faisal University, Al Hofuf 37912, Saudi Arabia; galjowf@kfu.edu.sa

**Keywords:** topical corticosteroids, steroid phobia, corticophobia, medication adherence, dermatology, cross-sectional study, Saudi Arabia

## Abstract

**Background/Objectives:** Topical corticosteroids (TCS) are a cornerstone of dermatological treatment for inflammatory skin conditions; however, irrational fear of their use known as corticophobia undermines adherence and worsens clinical outcomes. This study investigated the prevalence of TCS-related concern and phobic behaviors among the general population in Saudi Arabia and identified factors associated with TCS-related concern and phobic attitudes. **Methods**: A cross-sectional study was conducted between October and December 2025 using an online self-administered questionnaire distributed via social media platforms among a non-probability convenience sample across multiple geographic zones of Saudi Arabia. A total of 481 participants were enrolled. Descriptive statistics summarized demographic and clinical characteristics. Chi-square and Fisher’s exact tests were used to examine differences in proportions between categorical variables and TCS concern, while independent-samples *t*-tests and one-way ANOVA compared mean phobia scores across subgroups. **Results**: Of 481 participants, 254 (52.8%, 95% CI 48.3–57.2) expressed concern about TCS use. The predominant reason for refusing prescribed TCS was fear of side effects (93.5%). Phobic behaviors included fear of long-term use (54.2%) and fear of application to sensitive skin areas (63.0%). On the Likert phobia-scale item, 237 (49.8%) totally agreed they would use TCS if prescribed; a separate dichotomous behavioral-intention item, administered only to non-current users (n = 308), showed that 201 (65.3%) would accept TCS if prescribed and 107 (34.7%) would refuse. Concern was significantly more prevalent among females (58.1%, BH-adj *p* = 0.005), married participants (61.7%, BH-adj *p* = 0.010), and those refusing prescribed TCS (77.6%, BH-adj *p* < 0.001). Mean phobia scores (theoretical range 7–28) were significantly higher among females (20.43 ± 4.06 vs. males 18.84 ± 4.68, *p* < 0.001), participants with Diploma-level education (21.64 ± 3.12, *p* < 0.001 across education strata), widowed/divorced individuals (21.82 ± 3.57, *p* = 0.008), and residents of the Southern (20.47 ± 3.99) and Northern (21.40 ± 3.34) regions (*p* = 0.002 across regions). **Conclusions:** TCS-related concern was expressed by over half the participants in this social media-recruited sample, posing a substantial barrier to effective dermatological care. Side-effect concern was the most frequently reported reason for refusing prescribed TCS. In adjusted analyses, female sex was the most consistent independent correlate of TCS-related concern and higher phobia score; married status was independently associated with greater concern. These associations should be replicated in probability-based samples before subgroup-targeted interventions are designed. **Conclusions**: TCS-related concern was prevalent (52.8%) among adults in Saudi Arabia and represented a substantial barrier to dermatological care. Female sex and married status were independently associated with greater concern. Clinicians should proactively address TCS misconceptions during dermatological consultations to improve treatment adherence.

## 1. Introduction

Topical corticosteroids (TCS) represent one of the most frequently prescribed therapeutic agents in dermatology, serving as the cornerstone of treatment for a broad spectrum of inflammatory and autoimmune skin conditions, including atopic dermatitis, psoriasis, lichen planus, lichen simplex chronicus, discoid lupus erythematosus, vitiligo, and lichen sclerosis [1,2,3]. Available in various formulations, potencies, and vehicles, TCS efficacy is contingent upon selecting the appropriate preparation matched to the diagnosis, affected body site, and intended duration of therapy [2]. Since the introduction of hydrocortisone 1% cream as an over-the-counter agent in the United Kingdom in 1987, TCS have become widely accessible, facilitating their use and misuse beyond the scope of specialist prescription [4]. Unrestricted use has been associated with well-documented adverse effects, including cutaneous atrophy, striae, rosacea, acneiform eruptions, perioral dermatitis, and purpura; incidence decreases substantially when use is restricted to prescription-only frameworks [4,5].

Despite their established efficacy and safety profile when used appropriately, TCS phobia, defined as vague, unfavorable beliefs and irrational fear toward TCS, among patients or caregivers has emerged as a clinically significant barrier to effective dermatological care [6]. TCS phobia is directly associated with poor medication adherence, disease exacerbations, and unnecessary escalation to expensive biologics such as dupilumab or systemic immunosuppressants including methotrexate and cyclosporine [6,7,8,9,10,11,12,13]. Contributing factors include personal exposure to adverse effects, misinterpretation of prescribing information, and misinformation disseminated via social media and informal networks [14]. Addressing these factors through effective patient education requires a thorough understanding of their prevalence and determinants.

Published estimates of TCS phobia prevalence range widely from 31% to 95.7% depending on the study population, methodology, and tool used [15]. However, epidemiological data from Saudi Arabia remain limited, with only a small number of regional studies conducted in single cities or specific patient subgroups [16,17,18,19]. A cross-sectional assessment among a socially recruited sample covering diverse demographic regions and employing a validated phobia scale is lacking. This study aims to address this gap by determining the prevalence of TCS-related concern and phobic behaviors among the general population across Saudi Arabia, characterizing the beliefs and behaviors that associated with it, and identifying factors associated with higher TCS-related concern and phobic attitudes to inform targeted health education strategies.

## 2. Materials and Methods

### 2.1. Study Design and Setting

This was a cross-sectional, observational study conducted between October and December 2025 among the general population of Saudi Arabia. Data were collected via an online self-administered questionnaire distributed through social media platforms (WhatsApp, X [formerly Twitter], and Instagram). The study included participants from five major geographic zones of Saudi Arabia, broadly corresponding to the northern, southern, eastern, western, and central parts of the country. These zones were defined for the purpose of geographic distribution analysis and do not strictly correspond to the official administrative regions designated by the Saudi government; rather, they reflect the self-reported residential areas indicated by participants in the questionnaire.

### 2.2. Study Population and Sampling

Participants were eligible if they were Saudi nationals or long-term residents of Saudi Arabia aged 18 years or older and able to complete the questionnaire in Arabic. Individuals who identified themselves as healthcare professionals were excluded via a self-report screening item at the commencement of the questionnaire, to minimize professional knowledge bias. Questionnaires were retained if participants completed the core sociodemographic block and the primary TCS-concern item (n = 481). Module-specific denominators differ across the results because (i) the seven-item cumulative phobia score required complete responses to all seven items (n = 476); (ii) multivariable models list-wise excluded participants missing any covariate (logistic n = 481; linear n = 476); (iii) the ever-user module was conditional on current or previous TCS use (n = 192); and (iv) the usage-detail module was conditional on current use (n = 163). Convenience sampling was employed through across social media platforms.

### 2.3. Sample Size Calculation

The required sample size was calculated using the online Raosoft^®^ sample size calculator with an assumed population size of 35 million, a confidence interval of 95%, a margin of error of 5%, and an estimated TCS phobia prevalence of 50% (conservative estimate). The minimum required sample was 377 participants. A total of 481 valid responses were obtained, exceeding the calculated minimum.

### 2.4. Instruments

The phobia assessment items were adapted from established instruments used in the TCS literature, including the TOPICOP scale [10]. In addition, the selection of items was guided by findings from previously published cross-sectional studies and systematic reviews on TCS phobia [6,8,11]. Items were translated into Arabic by two bilingual investigators and back translated by an independent bilingual clinician; discrepancies were resolved by consensus. The instrument was pretested in 20 adults from the target population to confirm clarity and face validity.

The questionnaire was developed in Arabic and comprised four sections: (1) sociodemographic information (age, sex, marital status, education level, region); (2) TCS use history and current practices; (3) seven items assessing phobic attitudes and behaviors toward TCS, adapted from the TOPICOP scale [10] and previous TCS-phobia studies [6,8,11]; and (4) sources of information about TCS. Each of the seven phobia items was scored on a four-point Likert scale (1 = Totally Disagree, 2 = Neutral, 3 = Somewhat Agree, 4 = Totally Agree), as administered in the Arabic questionnaire. The willingness item (“If prescribed, I will use topical corticosteroids”) was reverse coded so that higher item scores consistently indicated greater phobic attitudes. The cumulative phobia score was computed as the sum of the seven items, with a theoretical range of 7–28 and higher values denoting stronger phobic attitudes. Because the instrument was adapted but not formally re-validated in Arabic, no diagnostic cut-off (low/moderate/high phobia) was imposed; the score is treated as a continuous measure of TCS-related concerns and phobic attitudes throughout. Questionnaires with any missing item on the phobia scale (n = 5) were excluded from score computation, yielding n = 476 with complete seven-item data. Internal consistency, calculated after reverse coding, was acceptable (Cronbach’s α = 0.73).

### 2.5. Data Analysis

Data was analyzed using IBM SPSS Statistics version 29.0 (IBM Co., Armonk, NY, USA). Descriptively, frequencies with percentages, means with standard deviations, and medians with interquartile ranges were used to characterize the study population. Confidence intervals of 95% were computed for key prevalence estimates. Differences in the distribution of categorical variables across levels of TCS concern were examined using Chi-square (χ^2^) test (or Fisher’s exact test where any expected cell frequency was less than 5); strength of association was quantified using Cramér’s V.

The distribution of the cumulative phobia score was assessed using the Shapiro–Wilk test; homogeneity of variance was assessed using Levene’s test. Mean phobia scores were compared between two groups using independent-samples *t*-tests and across three or more groups using one-way ANOVA. Effect sizes were reported as Cohen’s d for two-group comparisons, η^2^ for ANOVAs, and Cramér’s V for χ^2^ tests. The Benjamini–Hochberg procedure was applied jointly across the 16 primary tests to control the false-discovery rate.

For comparability with prior TCS-phobia studies using a binary endpoint [3,11], the primary outcome was dichotomized as TCS concern (“Yes” vs. “No/Neutral”). To identify factors independently associated with the outcomes, two multivariable models were fitted: (a) a binary logistic regression with TCS concern (Yes vs. No/Neutral) as the outcome, and (b) a multiple linear regression with the cumulative phobia score as the outcome. All covariates (age, sex, marital status, education, region, current TCS use, and primary information source) were selected a priori on the basis of plausibility from the prior TCS-phobia literature and entered simultaneously; no stepwise selection was performed. Adjusted odds ratios are reported for the logistic model; β coefficients (expressed as adjusted change in the cumulative phobia score in points, theoretical range 7–28, versus the reference category) are reported for the linear model. A two-sided *p*-value < 0.05 was considered statistically significant for all analyses.

### 2.6. Ethical Considerations

This study was approved by the Bioethics Committee of Scientific and Medical Research at the University of Jeddah (Bioethics Committee Registration Number: HAP-02-J-094; Application Number: UJ-REC-308; Approval Date: 29 December 2024), with all recruitment and data collection (October–December 2025) carried out prospectively within this approval period. Written electronic informed consent was obtained from all participants prior to completing the questionnaire. Participation was voluntary, and anonymity was assured.

## 3. Results

### 3.1. Sociodemographic Profile

A total of 481 participants were included in the analysis (Table 1). The most represented age group was 18–24 years (n = 225, 46.8%), followed by 35–44 years (n = 147, 30.6%) and 45–54 years (n = 64, 13.3%). Females comprised 66.9% (n = 322) of the sample. Most participants were single (55.7%), held a bachelor’s degree (58.2%), and resided in the Southern region (34.5%). Two willingness items were administered. First, on the four-point Likert phobia scale, 237 of 476 participants (49.8%) totally agreed with the statement “If prescribed, I will use TCS” (Table 2). Second, a separate dichotomous behavioral-intention item, administered only to participants not currently using TCS (n = 308), elicited “Yes” from 201 (65.3%, 95% CI 59.8–70.4) and “No” from 107 (34.7%); the latter group provided the denominator for the analysis of reasons for refusal in Table 1.

Among the 481 participants, 254 (52.8%, 95% CI 48.3–57.2) reported concern about TCS use, while a further 146 (30.4%, 95% CI 26.4–34.7) reported a neutral response, resulting in a total of 400 participants (83.2%, 95% CI 79.6–86.2) expressing some level of concern or neutrality. Among the 173 participants currently using TCS, the mean cumulative phobia score was 19.80 (SD 4.14); among the 303 not currently using TCS with complete phobia data, it was 19.96 (SD 4.45). For the full sample (n = 476 with complete seven-item phobia data), the mean phobia score was 19.90 (SD 4.34; 95% CI 19.51–20.29), and the median was 20 (interquartile range 17–23; observed range 7–28; theoretical range 7–28). The Shapiro–Wilk test indicated a statistically significant difference from normality (*p* < 0.001), reflecting mild deviation consistent with a ceiling effect; given the large sample size, parametric inference on subgroup means remains robust under the central limit theorem and was confirmed in non-parametric sensitivity analyses (Mann–Whitney U and Kruskal–Wallis), which yielded concordant conclusions. Complete sociodemographic data is presented in Table 1.

### 3.2. Reasons for Refusing Prescribed TCS

**Figure 1:**
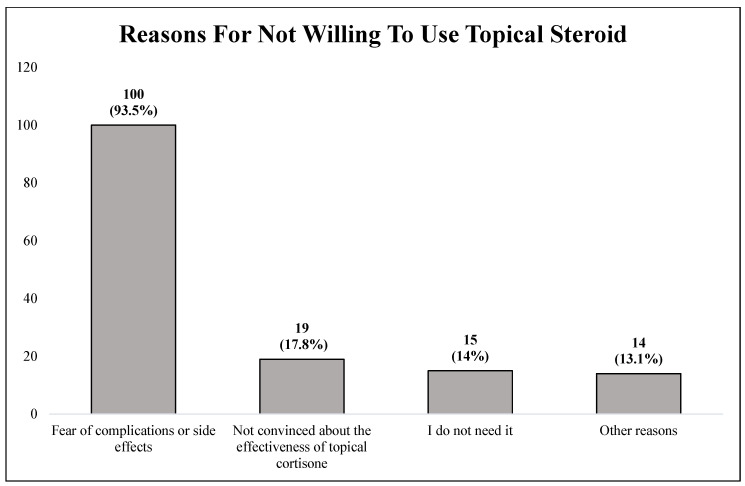
Reasons for unwillingness to use prescribed topical corticosteroids (TCS) among the 107 participants who reported they would not use TCS even if prescribed. Each bar shows the count and the corresponding percentage of the 107 participants. Multiple responses were permitted; therefore, percentages sum to more than 100%.

Among the 107 participants who stated they were unwilling to use TCS even if prescribed (Figure 1), the predominant reason was fear of complications or adverse effects (n = 100, 93.5%). Additional reasons included lack of confidence in TCS effectiveness (17.8%) and a belief that treatment was unnecessary (14.0%). Only 13.1% cited other reasons. The predominance of side-effect concern as the primary reason for non-use highlights the critical role of adverse-effect misconceptions in TCS phobia.

### 3.3. Sources of Information About TCS

**Figure 2:**
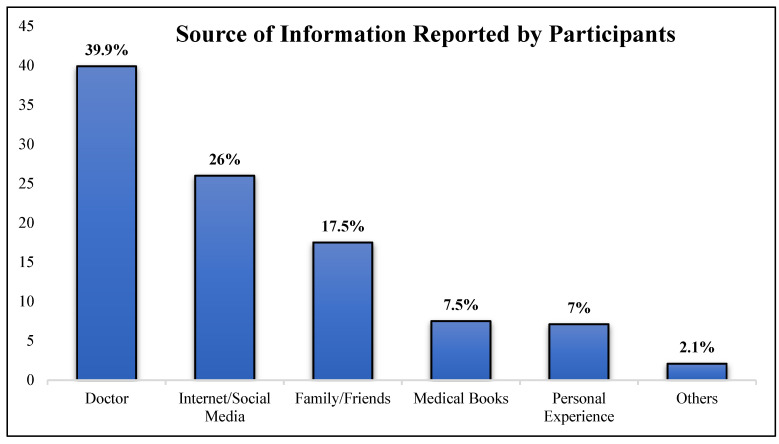
Sources of information about topical corticosteroids (TCS) (n = 481).

The primary source of information about TCS was healthcare providers, with 39.9% (n = 184) of participants citing doctors. However, 26% (n = 133) relied on the internet or social media, 17.5% (n = 80) on family or friends, 7.5% (n = 36) on medical books, and 7% (n = 33) on personal experience (Figure 2).

### 3.4. Treatment-Related Phobic Behaviors

Table 2 summarizes phobic behaviors related to TCS use. The most prevalent concern was fear of applying TCS to thin-skinned or sensitive areas such as the periorbital or genital region (62.6% totally agreed). Over half of participants (53.8%) were afraid of long-term use, and 40.3% feared applying large amounts or using TCS more than once daily. Despite these concerns, 49.5% stated they would use TCS if prescribed. More than a quarter (34.7%) preferred to delay treatment as long as possible, and 58.4% expressed the need for additional reassurance before initiating TCS therapy.

### 3.5. TCS Use Patterns, Concerns, and Reported Adverse Effects

Among all 481 participants, 254 (52.8%) expressed concern about TCS use, reflecting TCS-related worry rather than clinically defined phobia, while 146 (30.4%) reported a neutral response (Table 3).

Among the 192 ever-users (participants who reported current or previous TCS use), TCS were most commonly prescribed by dermatologists (n = 132, 68.8%). Most ever-users used TCS for less than two weeks (n = 109, 56.8%), and eczema and other skin diseases were the most common indications (both n = 68, 35.4%).

Among the 163 participants with complete usage data, TCS were applied once daily by the majority (n = 97, 59.5%), and most reported applying TCS to clean, dry skin (n = 135, 82.8%), and 106 (65.0%) perceived clinical improvement. However, 72 (44.2%) reported poor adherence. The main barriers to consistent use were insufficient hospital supply (n = 53, 32.7%) and high cost (n = 41, 25.3%). Additionally, 99 (60.7%) reported using non-corticosteroid alternatives, and 86 (52.8%) reported physician follow-up.

**Figure 3:**
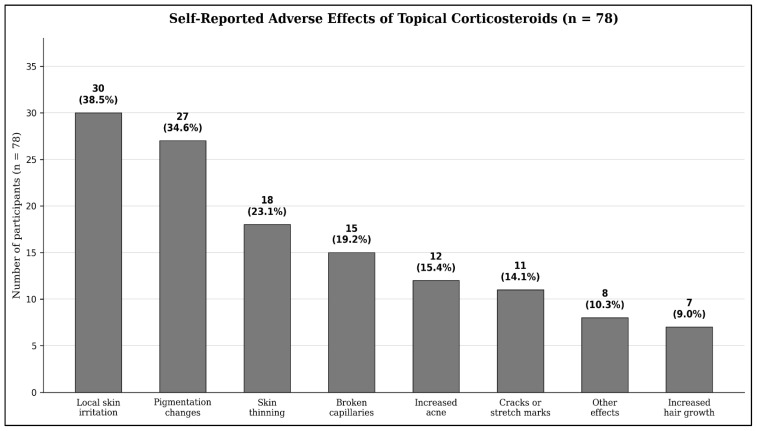
Self-reported adverse effects of topical corticosteroids (TCS) among the 78 ever-users who reported experiencing at least one adverse effect. Each bar shows the count and the corresponding percentage of the 78 participants. Multiple responses were permitted; therefore, percentages sum to more than 100%.

Among the 192 ever-users, 78 (40.6%) self-reported experiencing adverse effects, most commonly local skin irritation (38.5%), pigmentation changes (34.6%), skin thinning (23.1%), broken capillaries (19.2%), increased acne (15.4%), cracks or stretch marks (14.1%), other effects (10.3%), and increased hair growth (9.0%); however, these should be interpreted as perceived adverse effects, as clinical verification was not performed, and some symptoms may be attributable to the underlying skin condition rather than TCS use (Figure 3).

### 3.6. Factors Associated with TCS Concern

Table 4 displays bivariate associations between participant characteristics and TCS concern. Significant associations were identified for sex (*p* = 0.005), with females showing higher concern (58.1%); marital status (*p* = 0.010), with married individuals most concerned (61.7%); region (*p* = 0.033); education (*p* = 0.046), with bachelor-level participants showing higher concern; and behavioral intention to use TCS if prescribed (*p* < 0.001), with non-users refusing TCS showing markedly higher concern (77.6%). After Benjamini–Hochberg adjustment for multiple testing, the unadjusted age association (*p* = 0.041) did not retain significance (*p* = 0.054). Current TCS use and primary information source were not significantly associated with concern.

### 3.7. Factors Associated with TCS Phobia Scores

Table 5 presents associations between participant characteristics and mean phobia scores (theoretical range 7–28). Significantly higher scores were observed among females (20.43 ± 4.06 vs. males 18.84 ± 4.68, *p* < 0.001, BH-adj *p* = 0.001) and widowed/divorced individuals (21.82 ± 3.57, *p* = 0.008 across marital strata, BH-adj *p* = 0.016). Regional variation was significant (*p* = 0.002, BH-adj *p* = 0.006), with the highest scores in the Northern (21.40 ± 3.34) and Southern (20.47 ± 3.99) regions and the lowest in the heterogeneous “Other” category (17.10 ± 5.43). Education was significantly associated with phobia score (*p* < 0.001, BH-adj *p* = 0.001), with diploma-level participants reporting the highest mean (21.64 ± 3.12). Age was significantly associated with phobia score in the unadjusted analysis (*p* = 0.015, BH-adj *p* = 0.026), driven principally by the small 25–34-year subgroup (n = 17; 16.82 ± 5.38). Participants who expressed concern about TCS had significantly higher phobia scores (21.83 ± 3.18 vs. 17.69 ± 4.44, *p* < 0.001). Current TCS use and primary information source were not significantly associated with phobia score after BH correction.

Tukey HSD post hoc analysis of the significant ANOVAs clarified which group contrasts drove the omnibus differences. For age, the 25–34-year subgroup had a significantly lower mean phobia score than the 35–44 and 45–54-year strata (adjusted *p* < 0.05); no other age contrast reached significance. For region and education, the heterogeneous “Other” category had significantly lower scores than most other categories, reflecting its small and atypical composition (n = 29).

### 3.8. Multivariable Regression Analyses

Two multivariable models were fitted to identify factors independently associated with the outcomes (Table 6). The binary logistic regression for TCS concern (n = 481) had a Hosmer–Lemeshow goodness-of-fit *p* = 0.99 and McFadden pseudo-R^2^ = 0.061. The multiple linear regression for the cumulative phobia score (n = 476) had adjusted R^2^ = 0.061 (overall F = 2.46, *p* < 0.001). The maximum variance inflation factor was below 5, indicating no problematic multicollinearity in either model.

After simultaneous adjustment for the full covariate set, female sex was independently associated with both higher odds of concern (aOR 1.71, 95% CI 1.13–2.61, *p* = 0.012) and a higher phobia score (β = +1.59 points, 95% CI +0.70 to +2.48, *p* = 0.001). Married status was independently associated with higher odds of concern (aOR 2.02, 95% CI 1.08–3.78, *p* = 0.029) but not with the continuous phobia score. Diploma-level education was independently associated with a higher phobia score (β = +1.81, *p* = 0.029). Residence in the Southern region was borderline non-significant in both adjusted models (aOR *p* = 0.07; β *p* = 0.069). Notably, the unadjusted associations of age and primary information source did not retain independent significance in either model, indicating that their crude effects were partially confounded by other covariates.

## 4. Discussion

This cross-sectional study provides an assessment of TCS-related concerns and phobic behaviors among a convenience sample recruited via social media across multiple regions of Saudi Arabia. Our finding that 51.6% of participants expressed concern about TCS use reflects the burden of TCS-related apprehension and behavioral avoidance within this population; however, this figure should be interpreted with caution, as it is derived from a single self-reported item rather than a validated diagnostic instrument, and does not constitute a clinically defined prevalence of TCS phobia. Nonetheless, this proportion falls within the global prevalence range of 31–95.7% reported in a comprehensive review by Contento et al. (2021) [15]. This comparatively moderate prevalence may reflect the mixed composition of our sample, which included both TCS-naïve and experienced users, as well as participants with varying levels of dermatological exposure.

Multivariable regression analyses identified female sex, married status, and diploma-level education as the most consistent independent correlates of TCS-related concern and phobic attitudes. The persistence of the female–male difference (aOR 1.71 for concern; β = +1.59 points for the phobia score) consistent with international observations of greater TCS hesitancy among women and is plausibly mediated by greater dermatological help-seeking, differential exposure to dermatology-related social-media content, lack of education, fear of side effects, and misinformation [15,20]. Conversely, age and primary information sources, which appeared significant in unadjusted analyses, lost independent significance in both models, indicating that their crude associations were partially confounded by the demographic composition of these subgroups. The associations that survived Benjamini–Hochberg adjustment for multiple testing, sex, marital status, education, region, and behavioral intention to use TCS if prescribed for the concern outcome, and sex, marital status, education, region and age for the phobia-score outcome should be regarded as the most robust signals in these data, while findings that did not survive correction should be interpreted as exploratory.

The overwhelmingly predominant reason for declining prescribed TCS was fear of adverse effects (93.5%), in alignment with Magboul et al. (2025), who similarly identified safety concerns as the primary factor associated with of corticosteroid hesitancy in the western region of Saudi Arabia [21]. Patients frequently overestimate the risk of systemic absorption, skin thinning, dependency, and withdrawal syndromes, while underestimating the established benefit-to-risk ratio of appropriately used TCS. These misconceptions are known to result in suboptimal application practices, interruptions in therapy, and avoidance of effective treatment, ultimately perpetuating chronic skin disease [6,12].

The age signal in the unadjusted analysis (driven principally by the small 25–34-year subgroup; n = 17) did not retain independent significance in either multivariable model. This is partially consistent with findings by Alamri and Al Satti (2024), who reported higher phobia in individuals aged ≥ 56 years [22], and with Lin et al. (2025), who reported that younger women (<25 years) were particularly concerned about cosmetically conspicuous TCS side effects such as facial acne and hyperpigmentation [23], suggesting that age-related patterns in TCS-related concern may vary considerably by setting, sex, and indication. In our cohort, age associations should be regarded as exploratory and require confirmation in larger probability-based samples.

The significantly higher phobia scores among female participants (20.43 ± 4.06 vs. males 18.84 ± 4.68, *p* < 0.001) are similar to Choi et al.’s (2020) findings, who observed greater TCS hesitancy among women using the TOPICOP score [3]. This difference may reflect heightened cosmetic awareness, greater dermatology healthcare utilization, or differential exposure to beauty-focused social media content. Furthermore, widowed and divorced individuals demonstrated the highest unadjusted phobia scores (21.82 ± 3.57, *p* = 0.008), suggesting that reduced social support, a recognized correlate of health anxiety and medication non-adherence, may amplify corticosteroid-related fear, as discussed by Janowski et al. (2012) in the context of psoriasis [24].

The role of information sources was particularly notable. Although physicians were the most frequently cited source (38.3%), a substantial proportion of participants relied on social media and the internet (27.7%) or family and friends (16.6%). Participants who sourced health information from social media demonstrated numerically higher unadjusted phobia scores (20.42 ± 4.14), although this association did not retain significance after BH correction (BH-adj. *p* = 0.114) and did not survive multivariable adjustment. This finding should be interpreted with caution in the context of a cross-sectional design, which precludes determination of directionality. Individuals with pre-existing TCS-related concerns may preferentially seek information from social media, rather than social media exposure itself precipitating phobic attitudes. Longitudinal studies are therefore warranted to delineate the temporal relationship between health information-seeking behavior and the development of TCS-related phobic attitudes. Nonetheless, clinicians should remain attentive to the potential influence of unregulated online health information and address TCS-related misconceptions proactively during consultations.

Key phobic behaviors identified in our study including fear of large-quantity application (40.5%), long-term use (54.2%), and application to sensitive anatomical sites (63.0%) mirror findings by Li et al. (2017) in their systematic review of TCS phobia in atopic dermatitis [6]. These behaviors directly undermine TCS efficacy and contribute to the vicious cycle of undertreated skin disease and escalating therapeutic costs. Poor adherence (44.2%) in our cohort is similarly consistent with published evidence linking phobic concerns to medication non-adherence [6]. Structural barriers including inadequate hospital dispensing (32.7%) and high out-of-pocket cost (25.3%) compound this problem and signal a need for systemic healthcare improvements alongside patient education.

The self-reported perceived adverse effects among TCS users (n = 78) were dominated by local skin irritation (38.5%), pigmentation changes (34.6%), and skin thinning (23.1%), which are consistent with the known dermatological sequelae of prolonged or inappropriate TCS use as described by Coondoo et al. (2014) [5]; however, given the absence of clinical verification, these symptoms should be interpreted with caution as they may partially reflect manifestations of the underlying dermatological condition rather than being directly attributable to TCS use. Among participants who reported adverse effects, a notable proportion had obtained TCS through self-medication or non-specialist sources (17.7% of users combined). While a direct causal relationship between non-specialist prescribing and the occurrence of adverse effects cannot be established from these cross-sectional data, this observation is consistent with the broader literature suggesting that unsupervised TCS use may be associated with a higher risk of adverse outcomes [5]. Strengthening evidence-based prescribing practices and enhancing patient counseling at the point of dispensing represent clinically plausible strategies to minimize inappropriate TCS use, though further prospective research is needed to substantiate these recommendations within the Saudi healthcare context.

Regionally, participants from the Southern and Northern regions reported the highest phobia scores, while those in the heterogeneous “Other” geographic category scored lowest. The Southern-region association was borderline non-significant in adjusted models (*p* = 0.07). These observed differences likely reflect variations in access to specialist dermatological care, regional health literacy, and differential exposure to TCS misinformation campaigns, and should be confirmed in geographically stratified probability-based samples before being used to guide geographic targeting of educational initiatives.

### 4.1. Strengths and Limitations

This study has several strengths, including its relatively large sample size, inclusion of participants from all major regions of Saudi Arabia, and use of a structured questionnaire that enabled assessment of TCS-related beliefs, behaviors, and phobia scores in a nationally distributed sample. The study also examined both general concern and quantified phobia scores, allowing identification of demographic and behavioral subgroups with higher TCS-related concern. However, several limitations should be considered. The findings are associative rather than causal given the cross-sectional design, and convenience sampling through social media may have introduced selection bias, limiting representativeness among older adults, those with limited internet access, and those with lower health literacy. The sample skewed younger (18–24 years) and female, consistent with social media recruitment bias, further limiting generalizability to older male populations. Self-reported data are subject to recall, social-desirability, and measurement bias, and the absence of clinician-verified diagnoses, objectively measured adherence, and confirmation of prescribed regimens limits the precision of clinical outcomes. Excluding healthcare professionals improved homogeneity but prevented comparisons with medically informed respondents. Although internal consistency was acceptable (Cronbach’s α = 0.730), the phobia assessment was adapted rather than fully validated, with no factor analysis or test–retest reliability conducted, and the multivariable models explained modest variance (adjusted R^2^ = 0.061; McFadden pseudo-R^2^ = 0.061), indicating that unmeasured determinants such as personality, prior adverse experiences, disease severity, and anti-TCS media exposure likely contribute. The willingness-to-use item was administered only to non-current users (n = 308). Future studies should employ stratified random sampling and the fully validated Arabic TOPICOP scale to improve construct validity, measurement precision, and the external validity of TCS-related concern estimates in the Saudi population.

### 4.2. Implications for Practice and Future Research

The findings of this study carry meaningful implications for both clinical practice and health policy. Dermatologists and primary care physicians should routinely screen for TCS-related misconceptions during consultations, particularly among female patients and those reporting reliance on online health information. Structured patient education protocols, incorporating individualized counseling on the benefit-to-risk profile of TCS, written information leaflets, and visual aids tailored to health literacy levels, should be integrated into standard dermatological care. Physicians should adopt a shared decision-making communication approach, explicitly eliciting and addressing patient concerns regarding adverse effects at the point of prescription. Given the observed associations between social media use and TCS-related concern, healthcare authorities, national dermatological societies, and the Saudi Ministry of Health should consider developing regulated, culturally appropriate, evidence-based digital campaigns to counter unverified online narratives surrounding TCS use. From a research perspective, future studies should employ prospective and interventional designs to evaluate the effectiveness of targeted educational interventions on phobia reduction and treatment adherence. The adoption of the validated TOPICOP scale across future multicenter studies would enhance cross-study comparability and enable more precise risk stratification, and qualitative research exploring the lived experiences of patients with TCS phobia would provide deeper insight into the cultural, psychological, and social determinants of corticosteroid hesitancy in the Saudi Arabian context.

Several methodological limitations warrant explicit acknowledgement. First, although internal consistency of the seven-item phobia scale was acceptable (Cronbach’s α = 0.730), the instrument was adapted from the literature rather than formally re-validated in Arabic; future Saudi work should use the formally validated Arabic TOPICOP scale once available. Second, the multivariable models explained a modest proportion of the variance (adjusted R^2^ = 0.061 for the phobia score; McFadden pseudo-R^2^ = 0.061 for concern), indicating that additional unmeasured determinants such as personality traits, prior adverse-treatment experiences, dermatological diagnosis severity, and exposure to specific anti-TCS media campaigns likely play important roles. Third, the willingness-to-use item was administered only to non-current users (n = 308), restricting analyses involving this variable to that subsample. Fourth, the cross-sectional design precludes causal inference, and self-reported data are subject to recall and social-desirability biases.

## 5. Conclusions

This study indicates that TCS-related concern and phobic behaviors were common among participants in this online convenience sample, with over half expressing concern about TCS use; however, these findings should not be interpreted as nationally representative prevalence estimates, given the non-probability sampling strategy and the absence of a validated diagnostic instrument for TCS phobia. Side-effect concern was the most frequently reported reason for refusing prescribed TCS. In adjusted analyses, female sex was the most consistent independent correlate of both TCS-related concern and higher phobia score; married status was independently associated with greater concern, and diploma-level education with higher phobia score. Regional and age associations did not retain independent significance after multivariable adjustment and require confirmation in probability-based samples before subgroup-targeted interventions are recommended. Clinicians should proactively address TCS misconceptions during consultations, and national health authorities should develop culturally sensitive, evidence-based educational campaigns delivered across both clinical and digital platforms to promote safe and effective dermatological care. Future studies employing validated measurement tools such as the TOPICOP scale and representative probabilistic sampling frameworks are necessary before reliable national prevalence estimates of TCS phobia can be established in the Saudi population.

## Figures and Tables

**Table 1:** Sociodemographic and clinical characteristics of all participants (n = 481).

Variable	Category	Frequency (%)
**Age (years)**	18–24	225 (46.8%)
	25–34	19 (3.9%)
	35–44	147 (30.6%)
	45–54	64 (13.3%)
	55–65	26 (5.4%)
**Sex**	Female	322 (66.9%)
	Male	159 (33.1%)
**Marital Status**	Single	268 (55.7%)
	Married	196 (40.7%)
	Widowed/Divorced	17 (3.5%)
**Education Level**	Up to High School	101 (21.0%)
	Bachelor’s Degree	280 (58.2%)
	Diploma	39 (8.1%)
	Postgraduate (Master’s/PhD)	27 (5.6%)
	Other/Unclassified	34 (7.1%)
**Residence Area**	Southern Region	166 (34.5%)
	Eastern Region	107 (22.2%)
	Western Region	100 (20.8%)
	Central Region	64 (13.3%)
	Northern Region	10 (2.1%)
	Other Sub-regions	34 (7.1%)
**Currently using topical corticosteroids**	No	308 (64.0%)
	Yes	173 (36.0%)
**Behavioral intention to use TCS if prescribed (non-current users only, n = 308)**	Yes	201 (65.3%)
	No	107 (34.7%)

Behavioral intention to use TCS if prescribed was asked only of participants not currently using TCS (n = 308).

**Table 2:** Treatment-related phobic behaviors regarding topical corticosteroid use (n = 476 with complete responses).

Statement	Totally Disagree	Neutral	Somewhat Agree	Totally Agree
**I am afraid to apply a large quantity of topical corticosteroids or use it more than once a day**	48 (10.1%)	91 (19.1%)	144 (30.3%)	193 (40.5%)
**I am afraid of using the cream for a long time**	37 (7.8%)	69 (14.5%)	112 (23.5%)	258 (54.2%)
**I am afraid of applying cream to sensitive areas (eyes, genitals)**	29 (6.1%)	52 (10.9%)	95 (20.0%)	300 (63.0%)
**If prescribed, I will use topical corticosteroids (Likert; all participants, n = 476)**	24 (5.0%)	75 (15.8%)	140 (29.4%)	237 (49.8%)
**It is best to wait as long as possible before using topical corticosteroids**	76 (16.0%)	123 (25.8%)	111 (23.3%)	166 (34.9%)
**I rub cream into my hands before applying to the affected area**	123 (25.8%)	135 (28.4%)	97 (20.4%)	121 (25.4%)
**I need more reassurance about using topical corticosteroids**	38 (8.0%)	78 (16.4%)	80 (16.8%)	280 (58.8%)

Note: Responses were scored using the four-point Likert scale as administered (1 = Totally Disagree, 2 = Neutral, 3 = Somewhat Agree, 4 = Totally Agree). Total TCS phobia scores were calculated by summing the seven items, yielding a theoretical score range of 7–28; reverse coding was applied to the willingness item.

**Table 3:** Topical corticosteroids use patterns, concerns, and clinical outcomes among participants.

Parameter	Category	Frequency N (%)
***Among all participants (n = 481)***		
**Concern about TCS use**	No	81 (16.8%)
	Neutral	146 (30.4%)
	Yes	254 (52.8%)
***Among ever-users (current or previous TCS use, n = 192)***		
**Prescriber**	Dermatologist	132 (68.8%)
	Self-Medicated	17 (8.9%)
	Pharmacist	14 (7.3%)
	Non-Dermatologist	13 (6.8%)
	Family/Relative Advice	9 (4.7%)
	Other	7 (3.6%)
**Duration of Use**	<2 Weeks	109 (56.8%)
	2 Weeks–3 Months	44 (22.9%)
	3 Months–1 Year	17 (8.9%)
	>1 Year	22 (11.5%)
**Indication**	Eczema	68 (35.4%)
	Other Skin Diseases	68 (35.4%)
	Psoriasis	14 (7.3%)
	Vitiligo	9 (4.7%)
	Other	33 (17.2%)
**Experienced Adverse Effects**	No	114 (59.4%)
	Yes	78 (40.6%)
***Among current users with complete usage data (n = 163)***		
**Frequency of Application**	Once a Day	97 (59.5%)
	Twice a Day	49 (30.1%)
	Once a Week	3 (1.8%)
	Twice a Week	1 (0.6%)
	Other/As Needed	13 (8.0%)
**Applied to Clean, Dry Skin**	No	28 (17.2%)
	Yes	135 (82.8%)
**Perceived Improvement**	No	57 (35.0%)
	Yes	106 (65.0%)
**Adherence**	Poor (often non-committed)	72 (44.2%)
	Good (mostly committed)	91 (55.8%)
**Barriers to Use**	No Issues	47 (29.0%)
	Insufficient Hospital Supply	53 (32.7%)
	High cost	41 (25.3%)
	Both	6 (3.7%)
	Other	15 (9.3%)
**Use of non-TCS Alternatives**	No	64 (39.3%)
	Yes	99 (60.7%)
**Follow-up with Physician**	No	77 (47.2%)
	Yes	86 (52.8%)

**Table 4:** Differences in TCS concern across participant characteristics (n = 481).

Variable	Category	No/Neutral N (%)	Concern N (%)	*p*-Value	Cramér’s V	BH-adj. *p*
**Age (years)**	18–24	116 (51.6)	109 (48.4)	0.041	0.144	0.054
	25–34	12 (63.2)	7 (36.8)			
	35–44	61 (41.5)	86 (58.5)			
	45–54	23 (35.9)	41 (64.1)			
	55–65	15 (57.7)	11 (42.3)			
**Sex**	Female	135 (41.9)	187 (58.1)	**0.001**	0.146	**0.005**
	Male	92 (57.9)	67 (42.1)			
**Marital**	Single	144 (53.7)	124 (46.3)	**0.004**	0.150	**0.010**
	Married	75 (38.3)	121 (61.7)			
	Widowed/Divorced	8 (47.1)	9 (52.9)			
**Education**	Up to High School	57 (56.4)	44 (43.6)	**0.031**	0.149	**0.046**
	Bachelor’s	118 (42.1)	162 (57.9)			
	Diploma	18 (46.2)	21 (53.8)			
	Postgraduate	12 (44.4)	15 (55.6)			
	Other	22 (64.7)	12 (35.3)			
**Region**	Southern	68 (41.0)	98 (59.0)	**0.021**	0.166	**0.033**
	Eastern	60 (56.1)	47 (43.9)			
	Western	45 (45.0)	55 (55.0)			
	Central	30 (46.9)	34 (53.1)			
	Northern	2 (20.0)	8 (80.0)			
	Other	22 (64.7)	12 (35.3)			
**Currently using TCS**	No	149 (48.4)	159 (51.6)	0.550	0.027	0.586
	Yes	78 (45.1)	95 (54.9)			
Behavioral intention if prescribed *	Yes	125 (62.2)	76 (37.8)	**<0.001**	0.372	**<0.001**
	No	24 (22.4)	83 (77.6)			
**Information source**	Doctor	94 (51.1)	90 (48.9)	0.292	0.113	0.334
	Internet/Social Media	53 (39.8)	80 (60.2)			
	Family/Friends	38 (47.5)	42 (52.5)			
	Medical Books	21 (58.3)	15 (41.7)			
	Personal Experience	15 (45.5)	18 (54.5)			
	Other	6 (40.0)	9 (60.0)			

Note: Chi-square (or Fisher’s exact where any expected cell < 5) tested differences in proportions across levels of TCS concern. Bold = significant at α = 0.05. Cramér’s V: small ≈0.10, medium ≈0.30, large ≈0.50. Benjamini–Hochberg adjustment applied jointly across 16 primary tests in Table 4 and Table 5. * The behavioral-intention item was administered only to participants not currently using TCS (n = 308); it is distinct from the Likert willingness item in Table 2, which was administered to all participants.

**Table 5:** Cumulative phobia score across participant characteristics (n = 476).

Variable	Category	n	Mean (SD)	*p*-Value	Effect Size	BH-Adj. *p*
**Age**	18–24 y	222	19.65 (4.27)	**0.015**	η^2^ = 0.026	**0.026**
	25–34 y	17	16.82 (5.38)			
	35–44 y	147	20.35 (4.26)			
	45–54 y	64	20.55 (4.00)			
	55–65 y	26	19.92 (4.71)			
**Sex**	Female	318	20.43 (4.06)	**<0.001**	d = 0.37	**0.001**
	Male	158	18.84 (4.68)			
**Marital**	Single	264	19.40 (4.47)	**0.008**	η^2^ = 0.020	**0.016**
	Married	195	20.42 (4.13)			
	Widowed/Divorced	17	21.82 (3.57)			
**Education**	Up to High School	101	19.39 (4.69)	**<0.001**	η^2^ = 0.043	**0.001**
	Bachelor’s	280	20.14 (4.10)			
	Diploma	39	21.64 (3.12)			
	Postgraduate	27	19.93 (4.24)			
	Other	29	17.10 (5.43)			
**Region**	Southern	166	20.47 (3.99)	**0.002**	η^2^ = 0.039	**0.006**
	Eastern	107	19.72 (4.56)			
	Western	100	19.47 (4.39)			
	Central	64	20.45 (3.82)			
	Northern	10	21.40 (3.34)			
	Other	29	17.10 (5.43)			
**Currently using TCS**	No	303	19.96 (4.45)	0.683	d = 0.04	0.683
	Yes	173	19.80 (4.14)			
**Information source**	Doctor	184	19.84 (4.27)	0.093	η^2^ = 0.020	0.114
	Internet/Social Media	131	20.42 (4.14)			
	Family/Friends	78	20.15 (4.16)			
	Medical Books	35	17.94 (4.68)			
	Personal Experience	33	19.85 (5.08)			
	Other	15	19.53 (4.49)			

Note: Independent samples *t*-test (Welch’s correction for unequal variance) for two-group comparisons; one-way ANOVA for three or more groups. Bold = significant at α = 0.05. Cohen’s d: small ≈0.20, medium ≈0.50, large ≈0.80. η^2^: small ≈0.01, medium ≈0.06, large ≈0.14. Theoretical range of cumulative phobia score: 7–28 (four-point Likert × seven items).

**Table 6:** Multivariable regression analyses of factors associated with TCS concern (logistic, n = 467) and cumulative phobia score (linear, n = 465).

Predictor (Reference)	Concern (Logistic)		Phobia Score (Linear)	
	aOR (95% CI)	*p* Value	β (95% CI)	*p* Value
**Age (18–24 y)**				
25–34 y	0.88 (0.06–12.51)	0.92	−1.41 (−4.58, +1.77)	0.39
35–44 y	0.96 (0.52–1.77)	0.90	−0.11 (−1.36, +1.14)	0.86
45–54 y	0.95 (0.42–2.15)	0.91	−0.15 (−1.83, +1.53)	0.86
55–65 y	0.60 (0.21–1.67)	0.32	+0.46 (−1.77, +2.69)	0.68
**Sex (Male)**				
Female	**1.71 (1.13–2.61)**	**0.012**	**+1.59 (+0.70, +2.48)**	**0.001**
**Marital (Single)**				
Married	**2.02 (1.08–3.78)**	**0.029**	+0.74 (−0.52, +2.00)	0.25
Widowed/Divorced	1.17 (0.37–3.76)	0.79	+1.88 (−0.48, +4.24)	0.12
**Education (≤High School)**				
Bachelor’s	1.38 (0.77–2.49)	0.28	+0.36 (−0.64, +1.36)	0.48
Diploma	1.20 (0.64–2.25)	0.58	**+1.81 (+0.19, +3.42)**	**0.029**
Postgraduate	1.43 (0.59–3.49)	0.43	+0.73 (−1.14, +2.61)	0.44
Other	1.08 (0.41–2.86)	0.87	−0.39 (−1.72, +0.95)	0.57
**Region (Eastern)**				
Southern	1.93 (1.10–3.37)	0.07	+0.99 (−0.08, +2.07)	0.069
Western	1.52 (0.90–2.57)	0.12	−0.28 (−1.46, +0.91)	0.64
Central	1.52 (0.85–2.71)	0.16	+0.71 (−0.69, +2.11)	0.32
Northern	3.59 (0.67–19.27)	0.14	+0.55 (−2.33, +3.44)	0.71
Other	1.08 (0.41–2.86)	0.87	−0.39 (−1.72, +0.95)	0.57
**Currently using TCS (No)**				
Yes	1.15 (0.75–1.75)	0.53	−0.30 (−1.14, +0.54)	0.48
**Information source (Doctor)**				
Internet/Social Media	1.37 (0.84–2.24)	0.21	+0.16 (−0.83, +1.16)	0.75
Family/Friends	1.19 (0.67–2.10)	0.55	+0.11 (−1.06, +1.28)	0.85
Medical Books	0.98 (0.45–2.14)	0.97	−1.19 (−2.76, +0.39)	0.14
Personal Experience	1.29 (0.57–2.91)	0.54	+0.27 (−1.35, +1.90)	0.74
Other	1.62 (0.53–5.00)	0.40	−0.34 (−2.60, +1.93)	0.77

Note: aOR = adjusted odds ratio; β = unstandardized adjusted change in cumulative phobia score (theoretical range 7–28) versus the reference category. Bold = significant at *p* < 0.05. Logistic model: McFadden pseudo-R^2^ = 0.061, Hosmer–Lemeshow approx. *p* = 0.99. Linear model: adjusted R^2^ = 0.061, F = 2.46, *p* < 0.001.

## Data Availability

The data presented in this study is available on request from the corresponding author. The data is not publicly available due to ethical restrictions and privacy concerns related to sensitive health information.

## Abbreviations

The following abbreviations are used in this manuscript:

TCS	Topical Corticosteroids
SD	Standard Deviation
TOPICOP	Topical Corticosteroid Phobia
BH	Benjamini–Hochberg
